# A non-clinical randomised controlled trial to assess the impact of pharmaceutical care intervention on satisfaction level of newly diagnosed diabetes mellitus patients in a tertiary care teaching hospital in Nepal

**DOI:** 10.1186/s12913-015-0715-5

**Published:** 2015-02-12

**Authors:** Dinesh Kumar Upadhyay, Mohamed Izham Mohamed Ibrahim, Pranaya Mishra, Vijay M Alurkar

**Affiliations:** Discipline of Social and Administrative Pharmacy, School of Pharmaceutical Sciences, Universiti Sains Malaysia, Penang, Malaysia; Department of Pharmacology, Manipal College of Medical Sciences and Manipal Teaching Hospital, Pokhara, Nepal; College of Pharmacy, Qatar University, PO Box 2713, Doha, Qatar; American University of the Caribbean School of Medicine, 1 University Drive at Jordan Road, Cupecoy, St. Maarten; Department of Medicine, Manipal College of Medical Sciences and Manipal Teaching Hospital, Pokhara, Nepal

**Keywords:** Diabetes, Nepal, Pharmacist, Pharmaceutical care, Non-clinical randomised controlled trial, Satisfaction

## Abstract

**Background:**

Patient satisfaction is the ultimate goal of healthcare system which can be achieved from good patient-healthcare professional relationship and quality of healthcare services provided.

Study was conducted to determine the baseline satisfaction level of newly diagnosed diabetics and to explore the impact of pharmaceutical care intervention on patients’ satisfaction during their follow-ups in a tertiary care teaching hospital in Nepal.

**Methods:**

An interventional, pre-post non-clinical randomised controlled study was designed among randomly distributed 162 [control group (n = 54), test 1 group (n = 54) and test 2 group (n = 54)] newly diagnosed diabetes mellitus patients by consecutive sampling method for 18 months. Diabetes Patient Satisfaction Questionnaire was used to evaluate patient’s satisfaction scores at baseline, three, six, nine and, twelve months’ follow-ups. Test groups patients were provided pharmaceutical care whereas control group patients only received their usual care from physician/nurses. The responses were entered in SPSS version 16. Data distribution was not normal on Kolmogorov-Smirnov test. Non-parametric tests i.e. Friedman test, Mann-Whitney U test and Wilcoxon signed rank test were used to find the differences among the groups before and after the intervention (p ≤0.05).

**Results:**

There were significant (p < 0.001) improvements in patients’ satisfaction scores in the test groups on Friedman test. Mann-Whitney U test identified the significant differences in satisfaction scores between test 1 and test 2 groups, control and test 1 groups and, control and test 2 groups at 3-months (p = 0.008), (p < 0.001) and (p < 0.001), 6-months (p = 0.010), (p < 0.001) and (p < 0.001), 9-months (p < 0.001), (p < 0.001) and (p < 0.001) and, 12-months (p < 0.001), (p < 0.001) and (p < 0.001) follow-ups respectively.

**Conclusion:**

Pharmaceutical care intervention significantly improved the satisfaction level of diabetics in the test groups compare to the control group. Diabetic kit demonstration strengthened the satisfaction level among the test 2 group patients. Therefore, pharmacist can act as a counsellor through pharmaceutical care program and assist the patients in managing their disease. This will not only modify the patients’ related outcomes and their level of satisfaction but also improve the healthcare system.

## Background

Patient satisfaction has an important place in the healthcare system. Patient satisfaction can be obtained from a good patient-healthcare professional relationship, which influences patient health-related outcomes [1]. The measurement of patients’ satisfaction is the prominent method to describe how well the services are provided with a realistic indication of the quality of healthcare services, which reflects whether or not the patient has achieved his/her expectation with the provided services and also allows assessment of the healthcare professional performance [2,3]. Patient satisfaction can be viewed as “the extent of an individual’s experience compared with his or her expectations” [4] where expectation is “a reference point consumers use to assess their service experience” [2]. Patient satisfaction can only be achieved when patient perception towards healthcare services is optimistic, gratifying and meets their expectation. In Nepal about 90% of the population lives in rural areas where healthcare facilities are poor. Moreover, geographical division of the country isolated the rural areas from central areas and has hindered the development of transportation, communication and health facilities.

Most studies from developed countries have shown the impact of pharmaceutical care services on patients’ satisfaction [5-8] but studies have not given the attention in executing any kind of module for better patients’ understanding about their disease and its management which is directly linked with patients’ satisfaction. Moreover, studies conducted worldwide to assess the satisfaction level have not targeted the newly diagnosed diabetes patients whose expectation from healthcare system and satisfaction level might be different from those diabetics receiving the treatment since long time. Studies addressing the impact of pharmaceutical care services on patients’ satisfaction level are infancy in developing countries like Nepal where pharmaceutical care concept is still in the theoretical stage and pharmacists are still finding ways to participate in patient care. Therefore, the present study was conducted to assess the impact of pharmaceutical care intervention provided by a pharmacist on satisfaction level of newly diagnosed diabetes mellitus patients in Nepal.

## Methods

### Study design

The study was an interventional, pre-post non-clinical randomised controlled trial among the control group (CG), test 1 group (T1G) and test 2 group (T2G) with three treatment arms exploring the impact of pharmaceutical care intervention on satisfaction of newly diagnosed diabetes patients at the Manipal Teaching Hospital, Pokhara, Nepal for a period of 18 months (July 2010 to December 2011). The study was approved by the Research and Ethics Committee of Manipal Teaching Hospital, Nepal.

### Study population

The study population was the newly diagnosed type 1 and type 2 diabetes mellitus patients of aged 16 years and above. Pregnant women, mentally incompetent patients and patients not willing to participate in the study were excluded. Patients who did not come at their first follow-up were also excluded. Patients were requested to sign a written consent form as acceptance to participate in the research study. However, in case of minors, parental consent was sought and obtained.

### Sample size and sampling technique

Sample size was calculated by using a finite population correction formula [9]. Diabetes prevalence of 9% was taken as the calculation factor from previous studies [10,11]. The Z value was set at 1.96, with a 95% of confidence interval and 5% as margin of error. The calculated sample size was 125 patients. A drop-out margin of 30% was taken from previous studies [12,13] and added to the sample to achieve the final targeted sample size of 162 patients. The targeted sample size (162 patients) was achieved by a consecutive sampling method (based on time capsule frame) over 6 months duration (from July 2010 to December 2010) [14]. The randomisation of 162 patients was done by 1:1:1 in three parallel groups [15]: CG (n = 54), T1G (n = 54) and T2G (n = 54) without disturbing the sequence of randomisation. A total of ten patients (4 patients from control group and 3 patients from each test group) did not complete their first assessment follow-up (3-months) and therefore, further study was carried out with only 152 patients divided in to CG (n = 50), T1G (n = 51) and T2G (n = 51).

### Study tools

All the study tools and the questionnaire were prepared in the Nepali language due to language fluency and barriers to the English language among most of the patients visiting the hospital. Socio-demographic characteristics of the patients were documented in socio-demography form. Diabetes information booklet, diabetes complication chart, diabetic food chart were used as an educational materials to increase the patients’ awareness about diabetes and its management. A diabetic kit (including glass tubings, chart of human anatomy with circulatory system, daily medication calendar and calendar of antidiabetic medicines) was made especially for T2G (PC + Diabetic kit group) patients to explain about anatomical and physiological relationship of diabetes and its impact on physiological system. The purpose to use the diabetic kit only in T2G patients was to identify whether the extra demonstration of the kit brings any significant change in patients’ satisfaction scores between T1G and T2G.

### Formulation of Diabetes Patient Satisfaction Questionnaire

The theme and key words of Diabetes Patient Satisfaction Questionnaire (DPSQ) were extracted and compiled from different resources [2,3,16-19]. A total of 16 questions were constructed in English after an in-depth literature review related to patient’s satisfaction/dissatisfaction towards pharmacist, pharmacy-provided services and treatment satisfaction following Nepali translation (native language) of the questionnaire by a professional translator. Eight questions addressed the patient’s satisfaction/dissatisfaction to pharmacist related parameters like attitude and behaviour, professional relationship, concern about the disease, courtesy and respect, communication ability, contribution/support in diabetes care, ability to answer queries, and amount of time spent, while the rest 8 questions focused on information received from the pharmacist about the disease and medications by using various diabetes educational materials. The questionnaire was tested for its validity before using it in the study.

### Validation and scoring method of diabetes patient satisfaction questionnaire

The face and content validity of the questionnaire was done by one doctor (diabetologist), three pharmacists (2 PhDs and 1 master’s degree) and one nurse (master’s degree). Pilot study was carried out with thirty one newly diagnosed diabetes patients to test the questionnaire suitability and better patients understanding. These patients were excluded in the final study. The Cronbach’s α test for reliability of the questionnaire was 0.75 and internal consistency level was considered adequate. The scoring was done on 5-point Likert scale (very dissatisfied = 1, slightly dissatisfied = 2, neutral = 3, slightly satisfied = 4 and very satisfied = 5). The neutral score 48 (3x16 = 48) of patients was taken as the mid-point to classify the patients’ satisfaction level into ‘least satisfied’ (if median score ≤48) and more satisfied (if median score >48-80).

### Description of pharmaceutical care intervention among diabetes patients

The pharmaceutical care intervention was categorized into the intervention phase (phases I to III) and reinforcement phase (phases IV and V). The control group patients did not receive pharmaceutical care intervention from pharmacist and maintained on usual care obtained from physician/nurses throughout the study.

Patients from both the test groups received the information about meaning of diabetes, its types, sign and symptoms, reasons for high blood glucose, risk factors of diabetes, different short term (acute) and long term (chronic) complications of diabetes and role of pharmacological (antidiabetic medication) and non-pharmacological (lifestyle modification, diet and exercise) measures in management of diabetes. Besides this, test groups patients were also taught about how to administer insulin by using insulin pen or insulin syringe (if they were prescribed insulin as therapy) and trained regarding the use of glucometer for self-monitoring of blood glucose (SMBG) at home. Medication envelopes were used to dispense the prescribed medication (s) to the patients.

In addition to it, test 2 group patients were demonstrated about diabetic kit components such as glass tubings showing the change in the viscosity pattern of blood among diabetic and non-diabetic patients and the impact of increased sugar on the blood flow in different organ system with emphasis of blood coagulation and obstruction in blood flow in blood vessels in diabetes. Chart of human anatomy with circulator system was described to make the patients aware to locate the different organ system in the body and the supply of blood to these organs via blood vessels. Special focus was given to those organs which are mainly affected in diabetes like cardiac system, renal system, eye and brain. They were also explained about the location of pancreas and its role in diabetes. Daily medication calendar and antidiabetic medicine calendar were used to enhance the patients’ knowledge and compliance about the use of antidiabetic medication in diabetes management. After completing the intervention phase, patients were reinforced with the information in a systematic manner during their reinforcement phases, as patient education is not one-time process and it requires continuous reinforcement by healthcare professionals. All the patients (belonging to all three groups) were evaluated for their satisfaction using DPSQ questionnaire before and after disseminating the pharmaceutical care intervention at baseline and at three, six, nine and twelve months follow-ups respectively.

### Data collection and statistical analysis

The baseline patients’ satisfaction scores was evaluated from all three groups and the evaluation was repeated again at 3-months, 6-months, 9-months and 12-months using the same questionnaire. The responses were coded, entered and verified by using Statistical Package for Social Sciences (SPSS) version 16. Descriptive analysis such as frequency, mean, standard deviation, median and inter-quartile range (IQR) was calculated as per the requirement for data analysis. The scores were found to be skewed at p < 0.05 significance level when checked by using the Kolmogorov-Smirnov test. Therefore non-parametric tests were used as per the applicability to analyse the patients’ satisfaction scores. Friedman test, Mann-Whitney U test were used to find out the differences between dependent and independent variables within and between the groups before and after the interventions respectively. The Wilcoxon signed rank test was used for pre- and post-comparison within the groups. Post hoc analysis with Wilcoxon signed rank test was used to find where the significant differences actually occurred in the group at new p-value of ≤0.005 after Bonferroni adjustment. A significance level of ≤0.05 was used in all analyses.

## Results

### Socio-demography characteristics of study patients

There were 162 patients enrolled in the study. The mean age (in years) of the patients was 49.14 ± 12.56. Males were greater in number (n = 106, 65.43%). The mean ± sd of the body mass index (BMI) of patients was 27.60 ± 3.54 kg/m^2^_._ The median monthly income (in Nepali rupees, 1USD = 73.38 Nepali rupees) and inter-quartile range of the patients was 10,000 [(9,000)-(16,000)]. About 40.7% patients were unemployed, 25.9% businessman, 18.5% employed, 13.6% pensioner and 1.2% students in the study. The study found 30.9% patients either primary educated or secondary educated and, only 24% and 14.2% patients were non-educated and tertiary educated respectively. There were 92% patients of non-vegetarian food habits. Nearly 42.6% and 57.4% patients never had alcohol and smoking habits respectively while16.7% and 17.9% patients were found alcoholic and smoking every day. Moreover, only 24.1% and 6.8% patients were occasional and 16.7% and 17.9% were former alcoholic and smoker respectively. Type 2 diabetics were found more (n = 156, 96.3%) in the study. Majority of the patients (n = 133, 82.1%) did not have any family history of diabetes.

### Patients’ satisfaction with pharmacist and pharmaceutical care intervention at baseline and follow-ups

At baseline, patients were ‘neutral’ in response to pharmacist’s contribution/support (CG: 77.8%, T1G: 87% and T2G: 50%), pharmacist’s ability to answer the queries (CG: 72.2%, T1G: 51.9% and T2G: 55.6%) and amount of time spent by pharmacist (CG: 66.7%, T1G: 63% and T2G: 79.6%). A perceptible proportion of patients were not only ‘neutral’ but also ‘slightly dissatisfied’ and ‘very dissatisfied’ related to pharmaceutical care intervention. Patients were ‘neutral’ in response to medications used for diabetes care (CG: 87%, T1G: 66.7% and T2G: 59.3%) and counselling related to medicine use (CG: 51.9%, T1G: 55.6% and T2G: 46.3%) while ‘slightly dissatisfied’ to information related to medicine(s) side-effects (CG: 46.3%, T1G: 40.7% and T2G: 42.6%). Furthermore, the majority of the patients were ‘very dissatisfied’ with various diabetes educational material and its effectiveness such as the diabetic kit (CG: 50%, T1G: 72.2% and T2G: 83.3%), diabetes complication chart (CG: 64.8%, T1G: 77.8% and T2G: 81.5%), diabetes information booklet (CG: 94.4%, T1G: 94.4% and T2G: 92.6%), diabetes food chart (CG: 83.3%, T1G: 75.9% and T2G: 79.6%) and overall counselling and diabetes education (CG: 72.2%, T1G: 63% and T2G: 61.1%) in the diabetes awareness program. Successive educational intervention from pharmacist using various educational materials related to diabetes management improved patients’ satisfaction in subsequent follow-ups. Further details related to patients’ responses to satisfaction questionnaire are depicted in Table 1.

**Table 1:** **Patients’ satisfaction with pharmacist and pharmaceutical care intervention at baseline and follow-ups**

**Items**	**Groups**	**Control group n (%)**					**Test 1 group (PC group) n (%)**					**Test 2 group (PC + Diabetes kit group) n (%)**				
	**Scales**	**Baseline**	**3 month**	**6 month**	**9 month**	**12 month**	**Baseline**	**3 month**	**6 month**	**9 month**	**12 month**	**Baseline**	**3 month**	**6 month**	**9 month**	**12 month**
Pharmacist attitude and behaviour	VD	0	0	0	0	0	0	0	0	0	0	0	0	0	0	0
	SD	0	0	0	0	0	0	0	0	0	0	0	1 (1.9)	0	0	0
	N	8 (14.8)	0	11 (22)	8 (16)	6 (12)	5 (9.3)	0	0	0	0	3 (5.6)	0	0	0	0
	SS	37 (68.5)	36 (72)	31 (62)	31 (62)	34 (68)	38 (70.4)	8 (15.7)	7 (13.7)	5 (9.8)	13 (25.5)	40 (74.1)	9 (17.6)	8(15.7)	9 (17.6)	8 (15.7)
	VS	9 (16.7)	14 (28)	8 (16)	11 (22)	10 (20)	11 (20.4)	43 (84.3)	44 (86.2)	46 (90.2)	38 (74.5)	11 (20.4)	41 (80.4)	43 (84.3)	42 (82.3)	43 (84.3)
Pharmacist’s professional relationship	VD	0	0	0	0	0	0	0	0	0	0	0	0	0	0	0
	SD	0	0	0	0	0	0	0	0	0	0	0	1 (1.9)	0	0	0
	N	8 (14.8)	33 (66)	39 (78)	41 (82)	41 (82)	17 (31.5)	15 (29.4)	18 (35.3)	16 (31.4)	7 (13.7)	14 (25.9)	22 (43.1)	24 (47.0)	21 (41.1)	8 (15.7)
	SS	31 (57.4)	17 (34)	11 (22)	7 (14)	9 (18)	28 (51.9)	23 (45.1)	20 (39.2)	25 (49)	34 (66.7)	30 (55.6)	23 (45.1)	25 (49)	25 (49)	38 (74.5)
	VS	15 (27.8)	0	0	2 (4)	0	9 (16.7)	13 (25.5)	13 (25.5)	10 (19.6)	10 (19.6)	10 (18.5)	5 (9.8)	2 (3.9)	5 (9.8)	5 (9.8)
Pharmacist’s concern about patients’ disease	VD	0	0	0	0	0	0	0	0	0	0	0	0	0	0	0
	SD	0	0	0	0	0	0	0	0	0	0	0	0	0	0	0
	N	24 (44.4)	11 (22)	15 (30)	10 (20)	14 (28)	15 (27.8)	4 (7.8)	4 (7.8)	2 (3.9)	0	11 (20.4)	1 (1.9)	2 (3.9)	0	1 (1.9)
	SS	25 (46.3)	38 (76)	35 (70)	39 (78)	36 (72)	36 (66.7)	27 (52.9)	25 (49)	29 (56.9)	28 (54.9)	37 (68.5)	40 (78.4)	40 (78.4)	37 (72.5)	19 (37.2)
	VS	5 (9.3)	1 (2)	0	1 (2)	0	3 (5.6)	20 (39.2)	22 (43.1)	20 (39.2)	23 (45.1)	6 (11.1)	10 (19.6)	9 (17.6)	14 (27.4)	31 (60.8)
Pharmacist’s courtesy and respect	VD	0	0	0	0	0	0	0	0	0	0	0	0	0	0	0
	SD	0	0	0	0	0	0	0	0	0	0	0	0	0	0	0
	N	0	3 (6)	6 (12)	7 (14)	7 (14)	3 (5.6)	0	0	0	0	1 (1.8)	0	0	0	0
	SS	22 (40.7)	38 (76)	35 (70)	39 (78)	36 (72)	25 (46.3)	19 (37.2)	23 (45.1)	8 (15.7)	4 (7.8)	19 (35.2)	13 (25.5)	15 (29.4)	8 (15.7)	4 (7.8)
	VS	32 (59.3)	9 (18)	9 (18)	4 (8)	7 (14)	26 (48.1)	32 (62.7)	28 (54.9)	43 (84.3)	47 (92.1)	34 (63.0)	38 (74.5)	36 (70.6)	43 (84.3)	47 (92.1)
Pharmacist’s communication ability	VD	0	0	0	0	0	0	0	0	0	0	0	0	0	0	0
	SD	0	0	0	0	0	0	0	0	0	0	0	0	0	0	0
	N	6 (11.1)	4 (8)	4 (8)	7 (14)	6 (12)	2 (3.7)	0	0	1 (1.9)	0	3 (5.6)	1 (1.9)	0	0	0
	SS	43 (79.6)	44 (88)	46 (92)	43 (86)	34 (68)	41 (75.9)	33 (64.7)	35 (68.6)	28 (54.9)	30 (58.8)	48 (88.9)	39 (76.5)	43 (84.3)	40 (78.4)	34 (66.7)
	VS	5 (9.3)	2 (4)	0	0	0	11 (20.4)	18 (35.3)	16 (31.4)	22 (43.1)	21 (41.2)	3 (5.6)	11 (21.6)	8 (15.7)	11 (21.6)	17 (33.3)
Pharmacist’s contribution/support	VD	0	0	0	0	0	0	0	0	0	0	0	0	0	0	0
	SD	6 (11.1)	0	0	0	0	2 (3.7)	0	0	0	0	19 (35.2)	0	0	0	0
	N	42 (77.8)	15 (30)	22 (44)	28 (56)	32 (64)	47 (87.0)	5 (9.8)	2 (3.9)	4 (7.8)	3 (5.9)	27 (50)	2 (3.9)	2 (3.9)	1 (1.9)	0
	SS	6 (11.1)	35 (70)	27 (54)	22 (44)	18 (36)	5 (9.3)	30 (58.8)	34 (66.7)	31 (60.8)	32 (62.7)	8 (14.8)	42 (82.3)	40 (78.4)	40 (78.4)	30 (58.8)
	VS	0	0	1 (2)	0	0	0	16 (31.4)	15 (29.4)	16 (31.4)	16 (31.4)	0	7 (13.7)	9 (17.6)	10 (19.6)	21 (41.2)
Pharmacist’s ability to answer the queries	VD	0	0	0	0	0	0	0	0	0	0	0	0	0	0	0
	SD	0	0	0	0	0	0	0	0	0	0	0	0	0	0	0
	N	39 (72.2)	19 (38)	9 (18)	16 (32)	12 (24)	28 (51.9)	3 (5.9)	1 (1.9)	0	0	30 (55.6)	2 (3.9)	1 (1.9)	0	0
	SS	15 (27.8)	31 (62)	41 (82)	34 (68)	36 (72)	26 (48.1)	29 (56.8)	31 (60.8)	30 (58.8)	27 (52.9)	24 (44.4)	36 (70.6)	37 (72.5)	36 (70.6)	29 (56.8)
	VS	0	0	0	0	2 (4)	0	19 (37.2)	19 (37.2)	21 (41.1)	24 (47.0)	0	13 (25.5)	13 (25.5)	15 (29.4)	22 (43.1)
Amount of time spent by pharmacist	VD	0	0	0	0	0	0	0	0	0	0	0	0	0	0	0
	SD	0	0	0	0	0	0	0	0	0	0	0	0	0	0	0
	N	36 (66.7)	22 (44)	8 (16)	9 (18)	10 (20)	34 (63.0)	1 (1.9)	0	0	0	43 (79.6)	1 (1.9)	2 (3.9)	0	0
	SS	18 (33.3)	28 (56)	40 (80)	37 (74)	33 (66)	20 (37.0)	25 (49)	21 (41.1)	20 (39.2)	16 (31.4)	11 (20.4)	34 (66.7)	35 (68.6)	33 (64.7)	19 (37.2)
	VS	0	0	2 (4)	4 (8)	7 (14)	0	25 (49)	30 (58.8)	31 (60.8)	35 (68.6)	0	16 (31.4)	14 (27.4)	18 (35.3)	32 (62.7)
Effectiveness of diabetic kit in diabetes awareness	VD	27 (50)	36 (72)	35 (70)	35 (70)	37 (74)	39 (72.2)	30 (58.8)	28 (54.9)	26 (51)	25 (49))	45 (83.3)	0	0	0	0
	SD	18 (33.3)	14 (28)	11 (22)	10 (20)	11 (22)	8 (14.8)	15 (29.4)	13 (25.5)	12 (23.5)	10 (19.6)	5 (9.3)	0	0	0	0
	N	9 (16.7)	0	4 (8)	5 (10)	2 (4)	7 (13.0)	6 (11.8)	10 (19.6)	13 (25.5)	16 (31.4)	4 (7.4)	0	0	0	0
	SS	0	0	0	0	0	0	0	0	0	0	0	21 (41.2)	20 (39.2)	15 (29.4)	11 (21.6)
	VS	0	0	0	0	0	0	0	0	0	0	0	30 (58.8)	31 (60.8)	36 (70.6)	40 (78.4)
Effectiveness of diabetes complication chart	VD	35 (64.8)	30 (60)	24 (48)	19 (38)	27 (54)	42 (77.8)	0	0	0	0	44 (81.5)	0	0	0	0
	SD	14 (25.9)	17 (34)	13 (26)	20 (40)	18 (36)	6 (11.1)	0	0	0	0	6 (11.1)	0	0	0	0
	N	5 (9.3)	3 (6)	13 (26)	11 (22)	5 (10)	6 (11.1)	0	0	0	0	4 (7.4)	0	0	0	0
	SS	0	0	0	0	0	0	20 (39.2)	16 (31.4)	15 (29.4)	12 (23.5)	0	18 (35.3)	9 (17.6)	12 (23.5)	4 (7.8)
	VS	0	0	0	0	0	0	31 (60.8)	35 (68.6)	36 (70.6)	39 (76.4)	0	33 (64.7)	42 (82.4)	39 (76.5)	47 (92.2)
Effectiveness of diabetes information booklet	VD	51 (94.4)	27 (54)	22 (44)	20 (40)	28 (56)	51 (94.4)	0	0	0	0	50 (92.6)	0	0	0	0
	SD	3 (5.6)	9 (18)	15 (30)	13 (26)	18 (36)	2 (3.7)	0	0	0	0	3 (5.6)	0	0	0	0
	N	0	13 (26)	13 (26)	17 (34)	4 (8)	1 (1.8)	1 (1.9)	1 (1.9)	1 (1.9)	1 (1.9)	1 (1.8)	1 (1.9)	0	0	0
	SS	0	1 (2)	0	0	0	0	28 (54.9)	24 (47.1)	26 (51)	24 (47.1)	0	18 (35.3)	16 (31.4)	22 (43.1)	17 (33.3)
	VS	0	0	0	0	0	0	22 (43.1)	26 (51)	24 (47.1)	26 (51)	0	32 (62.7)	35 (68.6)	29 (56.9)	34 (66.7)
Effectiveness of diabetic food chart	VD	45 (83.3)	3 (6)	3 (6)	2 (4)	3 (6)	41 (75.9)	0	0	0	0	43 (79.6)	0	0	0	0
	SD	3 (5.6)	5 (10)	4 (8)	6 (12)	9 (18)	7 (13)	0	0	0	0	0	0	0	0	0
	N	4 (7.4)	15 (30)	26 (52)	26 (52)	27 (54)	6 (11.1)	0	0	0	0	7 (13)	0	0	0	0
	SS	2 (3.7)	27 (54)	17 (34)	16 (32)	11 (22)	0	2 (3.9)	3 (5.9)	1 (1.9)	1 (1.9)	4 (7.4)	3 (5.9)	3 (5.9)	5 (9.8)	1 (1.9)
	VS	0	0	0	0	0	0	49 (96.1)	48 (94.1)	50 (98)	50 (98)	0	48 (94.1)	48 (94.1)	46 (90.2)	50 (98)
Effectiveness of overall counselling and education programme	VD	39 (72.2)	29 (58)	28 (56)	30 (60)	24 (48)	34 (63.0)	0	0	0	0	33 (61.1)	0	0	0	0
	SD	14 (25.9)	16 (32)	19 (38)	15 (30)	22 (44)	14 (25.9)	0	0	0	0	13 (24.1)	0	0	0	0
	N	1 (1.8)	5 (10)	3 (6)	5 (10)	4 (8)	6 (11.1)	6 (11.8)	3 (5.9)	4 (7.8)	3 (5.9)	8 (14.8)	7 (13.7)	8 (15.7)	5 (9.8)	3 (5.9)
	SS	0	0	0	0	0	0	29 (56.8)	32 (62.7)	37 (72.5)	36 (70.6)	0	33 (64.7)	33 (64.7)	35 (68.6)	31 (60.8)
	VS	0	0	0	0	0	0	16 (31.4)	16 (31.4)	10 (19.6)	12 (23.5)	0	11 (21.6)	10 (19.6)	11 (21.6)	17 (33.3)
Medication used for diabetes treatment	VD	2 (3.7)	0	0	0	0	11 (20.4)	0	0	0	0	10 (18.5)	0	0	0	0
	SD	5 (9.3)	0	0	4 (8)	6 (12)	7 (13.0)	0	0	0	0	8 (14.8)	0	0	0	0
	N	47 (87.0)	11 (22)	22 (44)	23 (46)	30 (60)	36 (66.7)	20 (39.2)	14 (27.4)	12 (23.5)	10 (19.6)	32 (59.3)	21 (41.2)	18 (35.3)	9 (17.6)	6 (11.8)
	SS	0	38 (76)	26 (52)	23 (46)	14 (28)	0	3 (5.9)	4 (7.8)	3 (5.9)	3 (5.9)	4 (7.4)	14 (27.4)	8 (15.7)	4 (7.8)	1 (1.9)
	VS	0	1 (2)	2 (4)	0	0	0	28 (54.9)	33 (64.7)	36 (70.6)	38 (74.5)	0	16 (31.4)	25 (49)	38 (74.5)	44 (86.2)
Counselling related to medicines use	VD	8 (14.8)	0	0	0	0	7 (13.0)	0	0	0	0	13 (24.1)	0	0	0	0
	SD	17 (31.5)	0	6 (12)	10 (20)	1 (2)	11 (20.4)	0	0	0	0	11 (20.4)	0	0	0	0
	N	28 (51.9)	15 (30)	19 (38)	12 (24)	13 (26)	30 (55.6)	20 (39.2)	14 (27.4)	12 (23.5)	10 (19.6)	25 (46.3)	21 (41.2)	18 (35.3)	9 (17.6)	6 (11.8)
	SS	1 (1.8)	33 (66)	21 (42)	23 (46)	35 (70)	6 (11.1)	4 (7.8)	2 (3.9)	3 (5.9)	1 (1.9)	5 (9.3)	6 (11.8)	7 (13.7)	5 (9.8)	1 (1.9)
	VS	0	2 (4)	4 (8)	5 (10)	1 (2)	0	27 (52.9)	35 (68.6)	36 (70.6)	40 (78.4)	0	24 (47)	26 (50.9)	37 (72.5)	44 (86.2)
Information about side effects of medicines	VD	8 (14.8)	6 (12)	8 (16)	6 (12)	0	10 (18.5)	0	0	0	0	17 (31.5)	1 (1.9)	0	0	0
	SD	25 (46.3)	24 (48)	29 (58)	28 (56)	25 (50)	22 (40.7)	2 (3.9)	2 (3.9)	3 (5.9)	0	23 (42.6)	5 (9.8)	2 (3.9)	2 (3.9)	2 (3.9)
	N	21 (38.9)	20 (40)	12 (24)	16 (32)	25 (50)	18 (33.3)	32 (62.7)	19 (37.2)	18 (35.3)	15 (29.4)	9 (16.7)	19 (37.2)	17 (33.3)	18 (35.3)	15 (29.4)
	SS	0	0	1 (2)	0	0	4 (7.4)	17 (33.3)	29 (56.8)	30 (58.8)	35 (68.6)	5 (9.3)	26 (50.9)	32 (62.7)	31 (60.8)	32 (62.7)
	VS	0	0	0	0	0	0	0	1 (1.9)	0	1 (1.9)	0	0	0	0	2 (3.9)

VD = very dissatisfied, SD = slightly dissatisfied, N = neutral, SS = slightly satisfied, VS = very satisfied.

### Patient satisfaction scores at baseline, 3-months, 6-months, 9-months and 12-months follow-ups

Patients’ responses to satisfaction questionnaire were analysed using descriptive statistics and responses are presented as median score and inter-quartile range (IQR). Patients from the three groups were found ‘least satisfied’ to pharmacist-provided pharmaceutical care (median scores <48) at baseline. The test groups patients perceived a greater improvement in their satisfaction scores (median scores >48) and found ‘more satisfied’ in subsequent follow-ups compared to their baseline satisfaction level due to pharmaceutical care intervention. The variation in median satisfaction scores between T1G and T2G was due to the difference in their interventional approach (use of diabetic kit in T2G). However, the continuous detrimental in satisfaction scores of control group patients could be seen during follow-ups and finally they were found ‘least satisfied’ at twelve months follow-up (median score <48). Further details about the satisfaction scores of patients are depicted in Table 2.

**Table 2:** **Patients’ satisfaction scores at baseline, 3-months, 6-months, 9-months and 12-months follow-ups**

**Groups**	**Control group**	**Test 1 group (PC group)**	**Test 2 group (PC + Diabetic kit group)**
**Follow-ups**	**Median score (IQR)**	**Median score (IQR)**	**Median score (IQR)**
Baseline	44 [(42)-(46)]	45 [(42)-(46)]	43 [(41)-(46)]
3-months (1^st^ F/U)	50 [(48)-(51)]	66 [(64)-(69)]	68 [(66)-(70)]
6-months (2^nd^ F/U)	49 [(48)-(50)]	67 [(65)-(70)]	69 [(66)-(71)]
9-months (3^rd^ F/U)	49 [(48)-(50)]	68 [(66)-(70)]	69 [(68)-(73)]
12-months (4^th^ F/U)	47.74 [(47)-(48.25)]	68 [(66)-(70)]	73 [(70)-(75)]

### Comparison of patient satisfaction scores at baseline, 3-months, 6-months, 9-months and 12-months follow-ups within test groups (Test 1 group and Test 2 group)

The statistically significant differences were determined in patients’ satisfaction scores of the test groups on Friedman test due to pharmaceutical care intervention (Table 3).

**Table 3:** **Comparison of patients’ satisfaction scores at baseline, 3-months, 6-months, 9-months and 12-months follow-ups within test groups (Test 1 group and Test 2 group)**

**Groups**	**Test 1 group (PC group)**	**Test 2 group (PC + Diabetic kit group)**
**Follow-ups**	**Median score (IQR)**	**Median score (IQR)**
Baseline	45 [(42)-(46)]	43 [(41)-(46)]
3-months (1^st^ F/U)	66 [(64)-(69)]	68 [(66)-(70)]
6-months (2^nd^ F/U)	67 [(65)-(70)]	69 [(66)-(71)]
9-months (3^rd^ F/U)	68 [(66)-(70)]	69 [(68)-(73)]
12-months (4^th^ F/U)	68 [(66)-(70)]	73 [(70)-(75)]
p-value*	<0.001**	<0.001**

*Friedman test.

**Differences were significant at ≤0.05 level (2-tailed).

To find out where the actual differences occurred in each group on different occasions, post-hoc analysis with the Wilcoxon signed rank test was used. The differences in satisfaction scores were considered significant at p ≤ 0.005 level (2-tailed) after Bonferroni adjustment. In test 1 group, the statistically significant differences in patients’ satisfaction scores were noted between baseline and first follow-up (Z = -6.221, p < 0.001), baseline and second follow-up (Z = -6.227, p < 0.001), baseline and third follow-up (Z = -6.219, p < 0.001), baseline and fourth follow-up (Z = -6.210, p < 0.001) and, first and fourth follow-up (Z = -3.072, p = 0.002). On the other hand, in test 2 group, the significant differences were found between baseline and first follow-up (Z = -6.222, p < 0.001), baseline and second follow-up (Z = -6.331, p < 0.001), baseline and third follow-up (Z = -6.228, p < 0.001), baseline and fourth follow-up (Z = -6.117, p < 0.001), first and third follow-up (Z = -3.047, p = 0.002), first and fourth follow-up (Z = -5.522, p < 0.001), second and fourth follow-up (Z = -5.658, p < 0.001) and, third and fourth follow-up (Z = -4.487, p < 0.001).

### Comparison of patients’ satisfaction scores between test groups and, control group and test groups

Mann-Whitney U test was used to analyse the differences in satisfaction scores of patients between test groups and, control group and test groups. The test revealed the statistical significant differences in satisfaction scores of patients at three, six, nine and twelve months follow-ups between the groups. Further details about differences in satisfaction scores between the test groups and, control and test groups are mentioned in Table 4.

**Table 4:** **Comparison of patients’ satisfaction scores between test groups (Test 1 group and Test 2 group) and, control group and test groups**

**Median satisfaction scores (IQR)**						
**Follow-ups**		**Baseline**	**3-months (1** ^**st**^ **Follow-up)**	**6-months (2** ^**nd**^ **Follow-up)**	**9-months (3** ^**rd**^ **Follow-up)**	**12-months (4** ^**th**^ **Follow-up)**
**Groups**	**T1G**	45 (42)-(46)	66 (64)-(69)	67 (65)-(70)	68 (66)-(70)	68 (66)-(70)
	**T2G**	43 (41)-(46)	68 (66)-(70)	69 (66)-(71)	69 (68)-(73)	73 (70)-(75)
	**CG**	44 (42)-(46)	50 (48)-(51)	49 (48)-(50)	49 (48)-(50)	47.74 (47)-(48.25)
p-value*	**T1G / T2G**	0.096	0.008**	0.010**	<0.001**	<0.001**
	**CG / T1G**	0.618	<0.001**	<0.001**	<0.001**	<0.001**
	**CG / T2G**	0.179	<0.001**	<0.001**	<0.001**	<0.001**

*Mann-Whitney U test.

**Differences were significant at ≤0.05 level (2-tailed).

## Discussion

Consumer satisfaction is linked with the quality of services provided to them, which indicates whether the services fulfilled the consumers’ expectations or not. In the context of healthcare services, the patients’ expectation is to receive good quality care and to achieve better health-related outcomes. Being an important member of healthcare team, it is the prime responsibility of the pharmacist to provide good services to patients.

At baseline, patients from the three groups were found ‘slightly satisfied’ with the components indicating the pharmacist’s attitude and behaviour, professional relationship, concern and communication ability, and ‘very satisfied’ with courtesy and respect received from the pharmacist. However, patients were ‘neutral’ with regard to pharmacist contribution/support, ability to answer the queries, and time spent. Patients were found to be ‘slightly satisfied’ and ‘very satisfied’ with few components related to pharmacist at baseline this could be the result of informal introductory session (to make the further conversation more friendly) between pharmacist and patients at their first meeting before proceeding to patient evaluation. Patients were ‘very dissatisfied’ with the use of various educational materials like diabetic kit, complication chart, diabetes information booklet, diabetic food chart and overall counselling and education program. They were ‘neutral’ with regard to diabetes medication and medication counselling while ‘slightly dissatisfied’ with the information related to medicine side-effects [20]. There were no significant differences in satisfaction scores of patients among the three groups and they were ‘least satisfied’ at baseline to pharmaceutical services.

Because of increasing patient burden, physicians are hardly getting enough time to listen patients’ problems with compassion and explaining about their disease and medication use to them. This could also be one of the reasons for patients’ dissatisfaction, which is supported by a study where authors identified patients’ dissatisfaction with respect to their willingness to listen with sympathy and explaining their problems in the out-patient clinic [20]. Another study from Saudi Arabia also described the need of more attention with the patients receiving antidiabetic medication in order to improve their treatment satisfaction [21]. Therefore, being an important member of healthcare team and easily accessible to patients, pharmacist can assist the physicians in order to enhance patients’ awareness about their disease and proper use of medication in disease management. This combined effort of healthcare professionals may increase the patient satisfaction which was found more with patients of test groups than control group in present study.

In general, patient satisfaction improved significantly in all aspects related to the pharmacist and diabetes education. Most of the patients were found ‘very satisfied’ with the pharmacist’s courtesy and respect and, attitude and behaviour [16] at twelve months but a considerable number of patients were ‘slightly satisfied’ with the pharmacists’ professional relationship. For other components related to the pharmacist, patient satisfaction ranged between ‘slightly satisfied’ to ‘very satisfied’. Furthermore, most of the patients were ‘very satisfied’ with the use of the diabetic food chart, diabetes complication chart, medication counselling and diabetic medications [22] while a perceptible number of patients were ‘slightly satisfied’ with the information related to medication side-effects. Similarly, patients achieved their goal of diabetic nutrition and medicines through diabetes educational program and hence improved patient satisfaction [19]. Patient satisfaction related to other components of diabetes education and counselling ranged between ‘slightly satisfied’ to ‘very satisfied’. The median satisfaction scores of the test groups were significantly greater than their baseline median satisfaction scores. Mann-Whitney U test identified the significant differences in satisfaction scores between the test groups due to differences in their interventional approach throughout the study period.

Comparing the present findings from previous studies related to patients’ satisfaction, one study from Brazil described the overall patient satisfaction with regard to pharmacy and pharmaco-therapeutic services [8]. Moreover, another study also explained the impact of pharmacist provided education on diabetes, lifestyle modification and pharmaco-therapeutic counselling which improved patients’ clinical and quality of life outcomes and, hence patients’ satisfaction [7]. In a community-based study where pharmacist-provided disease-related education using disease information leaflets and medication counselling upgraded the satisfaction level of hypertensive patients [17]. Importance of the pharmaceutical care program was also indicated from one randomised controlled trial where authors revealed the improvements in patient satisfaction with regard to clinical pharmacist-provided care and provision of medication information [6]. Strengthening the present finding with one Canadian study highlighted patient satisfaction with courtesy of the pharmacist, written information about the detailed care plan for the disease, medication use and its side effects at community pharmacies [23].

Although, there were slight improvements in satisfaction scores of the control group patients initially due to doctor- and nurse-provided care but a decreasing trend in satisfaction scores of the patients could be noticed in subsequent follow-ups. The consistent decreasing trends in satisfaction scores of control group patients in each follow-up indicates that the patients did not achieve their desired expectation and they were still facing problems in coping the disease.

## Conclusion

Pharmaceutical care intervention significantly improved the patients’ satisfaction in both the test groups compared to the control group throughout the study. Pharmacist involvement in patient care through pharmaceutical care services increased the level of patient’s satisfaction with all the aspects related to pharmacist and provided care. Intervention was more pronounced in improving patients’ satisfaction with regard to pharmacist’s courtesy and respect, attitude and behaviour, use of diabetic food chart, complication chart, medication counselling and medication used for diabetes care. It was noticed that the improvements were more prominent among test 2 group patients because of the demonstration of the diabetic kit. Therefore, the present finding indicates that the pharmacists can act as a counsellor through pharmaceutical care program that will not only improve the healthcare system but also modify the patients’ related outcomes and hence increase the satisfaction level of patients by helping them in managing their disease.

### Limitations of the study

The present study also had a few limitations. Diabetes patients were selected from only one hospital of the Kaski district in western Nepal and hence the study findings may not be able to generalize to the entire diabetes population of the country. A repeat measurement bias was expected since the same self-administered questionnaire was given to the participants. To limit this bias, however, patients were asked to complete the questionnaires in the presence of researcher.
